# Platinum-Based Regimens Are Active in Advanced Pediatric-Type Rhabdomyosarcoma in Adults and Depending on HMGB1 Expression

**DOI:** 10.3390/ijms24010856

**Published:** 2023-01-03

**Authors:** Nadia Hindi, Jaime Carrillo-García, Elena Blanco-Alcaina, Marta Renshaw, Pablo Luna, José Durán, Natalia Jiménez, Pilar Sancho, Rafael Ramos, David S. Moura, Javier Martín-Broto

**Affiliations:** 1 Health Research Institute Fundación Jiménez Díaz (IIS-FJD, UAM), 28040 Madrid, Spain; 2 Medical Oncology Department, University Hospital General de Villalba, 28400 Madrid, Spain; 3 Medical Oncology Department, University Hospital Fundación Jiménez Díaz, 28040 Madrid, Spain; 4 Institute of Biomedicine of Seville (IBIS), HUVR-CSIC-University of Seville, 41013 Seville, Spain; 5 CIBERONC, Instituto de Salud Carlos III, 28029 Madrid, Spain; 6 Medical Oncology Department, University Hospital Son Espases, 07210 Palma, Spain; 7 Medical Oncology Department, San Vicente de Paúl Hospital, Heredia 40101, Costa Rica; 8 Medical Oncology Department, University Hospital Virgen del Rocío, 41013 Seville, Spain; 9 Pathology Department, University Hospital Son Espases, 07210 Palma, Spain

**Keywords:** rhabdomyosarcoma, BOMP-EPI, cisplatin, HMGB1, HMGB2, HMGA2

## Abstract

Rhabdomyosarcoma (RMS) in adults is a rare and aggressive disease, which lacks standard therapies for relapsed or advanced disease. This retrospective study aimed to describe the activity of BOMP-EPI (bleomycin, vincristine, methotrexate and cisplatin alternating with etoposide, cisplatin and ifosfamide), an alternative platinum-based regimen, in adult patients with relapsed/metastatic RMS. In the study, 10 patients with RMS with a median age at diagnosis of 20.8 years and a female/male distribution of 6/4 received a mean of 2.5 cycles of BOMP-EPI. The best RECIST response was a complete response in 1/10 (10%) patients, a partial response in 5/10 (50%), stable disease in 3/10 (30%) and progression in 1/10 (10%). With a median follow-up in the alive patients from the start of therapy of 30.5 months (15.7–258), all patients progressed with a median progression-free survival of 8.47 months (95% CI 8.1–8.8), and 7/10 patients died with a median overall survival of 24.7 months (95% CI 13.7–35.6). BOMP-EPI was an active chemotherapy regimen in adults with pediatric-type metastatic RMS, with outcomes in terms of survival that seem superior to what was expected for this poor-prognosis population. Low HMGB1 expression level was identified as a predictive factor of better response to this treatment.

## 1. Introduction

Rhabdomyosarcoma (RMS) is a high-grade malignant neoplasm derived from the primitive mesenchymal cells with a propensity for myogenic differentiation, which can be developed in any part of the body. RMS is the most common soft tissue sarcoma (STS) in children, constituting 50% of sarcoma cases in childhood [1,2,3]. RMSs are subdivided into four subtypes, namely embryonal (ERMS), alveolar (ARMS), sclerosing and pleomorphic, according to the 2020 WHO classification, based on different histological, genetic and clinical features. ERMS is the most frequent subtype (60% of the cases), typically affecting children below 10 years of age and showing favorable outcomes. ERMS is characterized by accumulating copy number alterations and RAS pathway mutations. ARMS is the most undifferentiated and aggressive subtype (20% of the cases), commonly affecting adolescent and young adults, and it harbors the *PAX-FOXO1* fusion (*PAX3* or *PAX7*). Sclerosing RMS (10% of cases) is associated with an abundant hyaline matrix, affecting children and adults. Pleomorphic RMS is a highly aggressive subtype (10% of cases), affecting adults. 

Stage (nodal involvement, distant metastasis), location, size, disease volume after surgery (classified in the intergroup rhabdomyosarcoma study (IRS) groups), histologic subtype, age and the presence of specific molecular rearrangements are the main prognostic factors. Currently, the upfront treatment of RMS consists in a multimodal therapy, which includes surgical resection, systemic therapy based on intensive multidrug regimens and radiotherapy. According to the last 2005 EpSSG RMS stratification, first-line chemotherapy includes vincristine and actinomycin (VA) for the low-risk group, adding ifosfamide (IVA) for standard and high-risk groups and doxorubicin for the very high-risk group (IVADo). This multimodal therapy has shown overall survival rates over 90% in patients with a low-risk localized disease, but only of 21% or 30% in patients with a metastatic or recurrent disease, respectively [4,5]. Age is a known prognostic factor, being the prognosis of adolescents, young adults (AYA) and poorer adult patients, with more than half of patients succumbing to the disease [6]. In the case of adult patients with metastatic disease, the outcome is especially poor, with reported median overall survival slightly longer than 1 year. Patients with a metastatic or relapsed disease do benefit from chemotherapy, some patients with long disease-free intervals, but more than 70% of them will eventually relapse/progress and die from the disease [7]. The number of systemic options in advanced RMS is limited, and there is no consensus for the second and subsequent lines of therapy [8,9].

Platinum-based regimens have shown activity in pediatric patients with RMS enrolled in small clinical trials, with overall response rates between 28% and 39% [10,11,12,13], but there is a lack of data regarding adult patients. In preclinical studies, cisplatin has shown to be active in the in vitro and in vivo xenograft models of RMS and Ewing sarcoma [14]. Platinated compounds exert their cytotoxicity by binding covalently to the nucleophilic N7-sites of the purine bases DNA of the same strand (intrastrand crosslinks) or both strands (interstrand crosslinks (ICL)). These crosslinks are extremely cytotoxic, especially in proliferating cells, because they inhibit vital cellular processes such as replication and transcription and induce cell cycle arrest. In addition, DNA containing ICLs induce double-strand DNA breaks (DSB), which activate the DNA damage response (DDR) for repairing them or otherwise induce cell death by apoptosis [15,16,17]. 

DNA-platinum adducts can be recognized by the nonhistone chromosomal high-mobility group (HMGs) proteins, which are involved in the maintenance and functional regulation of DNA, such as replication, recombination, transcription and DNA repair [18,19]. HMGs proteins are classified into HMG-AT-hook (HMGA), HMG-box (HMGB) and HMG nucleosome-binding (HMGN) families, according to the structure of their DNA-binding domain and their substrate-binding specificity. High levels of some HMGs proteins, such as HMGA2, HMGB1 and HMGB4, can sensitize tumors to cisplatin by protecting DNA lesions from DNA repair machinery accession [20,21]. High levels of HMGA1 protein also sensitize to cisplatin by diminishing BRCA1 expression [22]. However, HMGA2 and HMGB1 overexpression in osteosarcoma cell lines induce resistance to cisplatin by inducing autophagy [23,24]. HMGB3 overexpression also induces resistance to cisplatin in ovarian cancer cells by ATR and CHK1 downregulation [25].

This study aims to describe the activity of alternative platinum-based regimes (specifically BOMP/EPI) in AYA and adult patients with metastatic/relapsed RMS and identify low HMGB1 expression level as a predictive factor of better response to this treatment.

## 2. Results

### 2.1. Clinical and Demographic Characteristics

In this study, 10 patients with RMS with a median age at diagnosis of 20.8 years (17–44) were treated with BOMP-EPI at a median age of 22.6 years (18–46). There was a female predominance (60%/40%), and the most frequent subtype was alveolar RMS (6/10). In 60% of cases, primary tumors arose in the head and neck. All patients had a high- or very high-risk disease based on EpSSG classification and five patients had metastatic disease at diagnosis. All except for one patient underwent surgery for the primary tumor (Table 1).

Three patients with metastatic disease at diagnosis received BOMP-EPI as their upfront therapy and seven patients with relapse (five metastatic and two locally advanced, with a median disease-free survival from diagnosis of 18.5 months (95% CI 6–31)) received BOMP-EPI after pretreatment with alkylating agents (IVA or IVADo in four patients, alternating VAC and IE in two patients or EPI/IFOS in one patient) (Table 2). Three patients received rechallenges of BOMP-EPI at progression (Table 3). The cause for the discontinuation of therapy was progression in five cases, complete remission in combination with local therapy in three cases, and toxicity in two patients. Seven patients experienced grade ≥ 3 neutropenia, with two patients with febrile neutropenia, but there were no toxic deaths. No pulmonary adverse events were described.

One patient (10%) showed a complete response (CR), five patients (50%) showed a partial response (PR), three patients (30%) showed a stable disease (SD) and one patient (10%) showed progression, according to the RECIST response (Table 3). At a median follow-up of 30.5 months (15.7–258) from the start of therapy, all alive patients had progressed with a median progression-free survival (PFS) of 8.47 months (95% CI 8.1–8.8) and 7/10 patients had died with a median overall survival (OS) of 24.7 months (95% CI 13.7–35.6). One-year PFS and OS were 40% and 80%, respectively. No differences regarding histologic subtype were found in median PFS (8.47 months (95% CI 1–15.9) in alveolar RMS vs. 8.34 months (95% CI 5.6–11) in embryonal/spindle cell RMS, *p* = 0.61) or OS (26.2 months (95% CI 13.7–35.6) in alveolar RMS vs. 10.8 months (95% CI 1–35.8) in embryonal/spindle cell RMS, *p* = 0.34). Three patients received a rechallenge of BOMP-EPI at progression after stopping therapy while in response (two patients stopped therapy because of adverse effects after two and three cycles, respectively, and the third patient completed six cycles). At rechallenge, two of three obtained a new PR, with disease control for 15.8 months in one patient and without evidence of disease after 229 months of follow-up in another patient. More in detail, this last patient had been diagnosed from a prostatic embryonal rhabdomyosarcoma with pulmonary metastasis when he was 42 years old. After diagnostic biopsy, he started induction therapy with BOMP-EPI, receiving six cycles, achieving a complete radiological response. Surgery (prostatectomy) was performed. After 29 months of follow-up, a disease relapse on the proximal femur was diagnosed. Induction chemotherapy with BOMP-EPI was restarted, receiving five cycles, achieving again a radiological complete response. The patient then underwent radical surgery, with a hip replacement. After surgery, he received high-dose chemotherapy with autologous stem-cell rescue. He is free of disease after 19 years of follow-up.

### 2.2. HMGB1 as a Predictive Biomarker for BOMP-EPI Treatment in RMS

**Figure 1:**
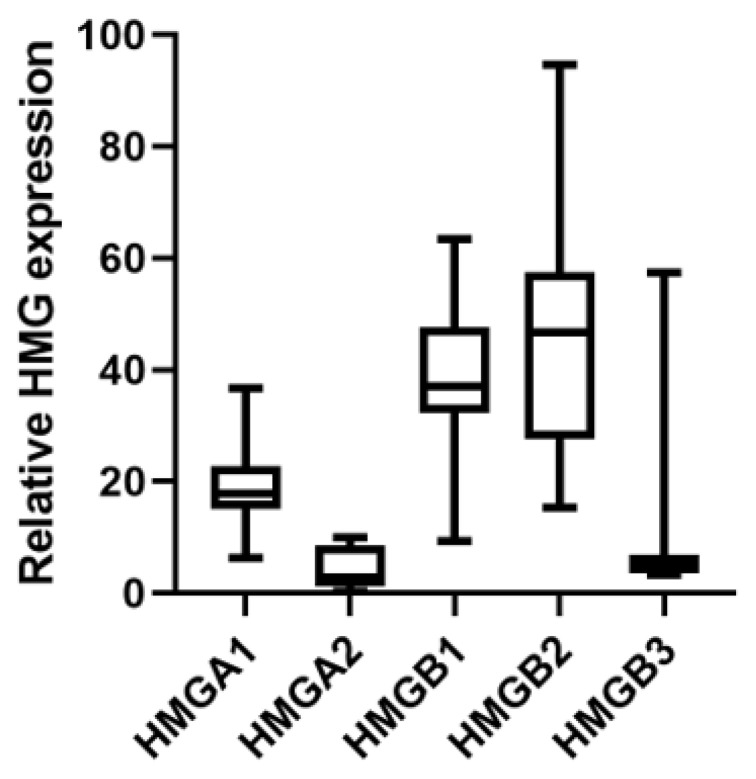
HMGA1, HMGA2, HMGB1, HMGB2 and HMGB3 gene expression in tumors of patients with RMS treated with BOMP/EPI. HMGA1, HMGA2, HMGB1, HMGB2 and HMGB3 gene expression in biopsies of eight patients with RMS treated with BOMP/EPI were analyzed with the EdgeSeq Oncology Biomarker Panel. The total counts of each gene were normalized by GAPDH counts in each sample and the mean value (*1000) for each gene was represented.

**Figure 2:**
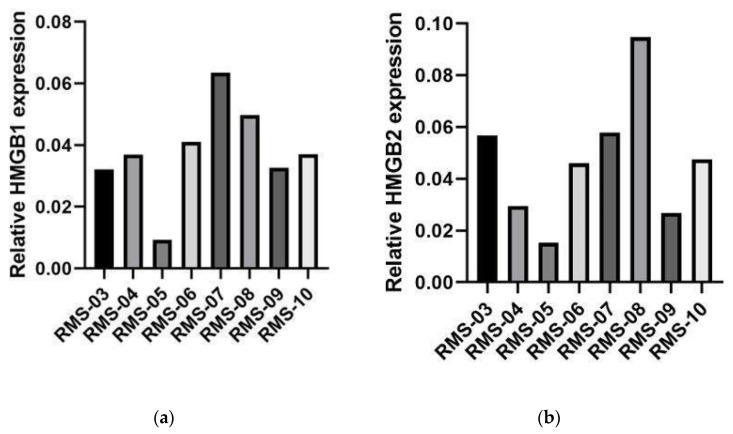

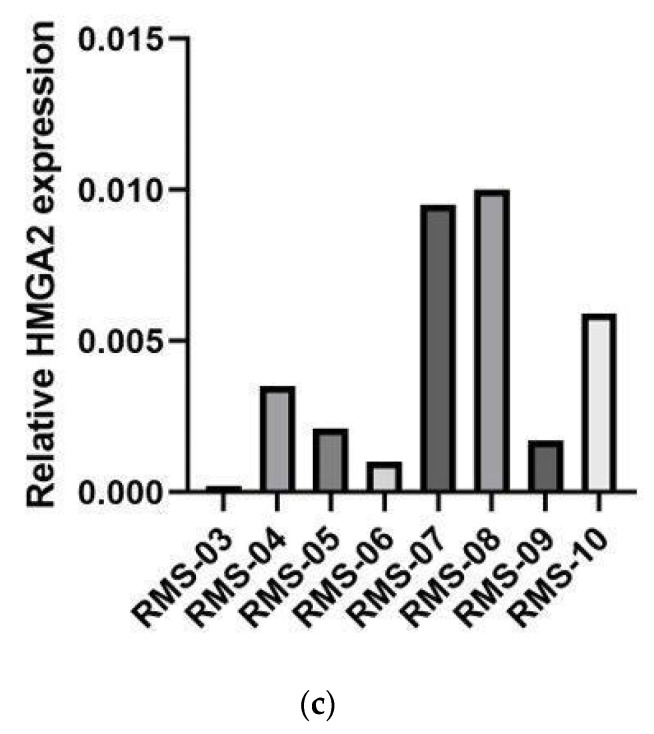
HMGA2, HMGB1 and HMGB2 gene expression in tumors for patients with RMS treated with BOMP/EPI. HMGB1 (**a**), HMGB2 (**b**) and HMGA2 (**c**) gene expressions in biopsies of seven patients with RMS treated with BOMP/EPI were analyzed with the EdgeSeq Oncology Biomarker Panel. The total counts of HMGA2, HMGB1 and HMGB2 obtained for each sample were normalized by GAPDH counts. RMS-03, RMS-07, RMS-08 and RMS-09: alveolar rhabdomyosarcoma tumor samples. RMS-04, RMS-05 and RMS-06: embryonal rhabdomyosarcoma tumor samples. RMS-10: spindle cell rhabdomyosarcoma tumor sample.

Gene expression of different HMGA and HMGB genes were analyzed in tumor samples from patients with RMS before BOMP-EPI treatment as the upfront therapy (RMS-05, RMS-06 and RMS-07), after relapse (RMS-04 andRMS-08) with other therapies (IVA/V or EPI-IFOS) or before relapse (RMS-03, RMS-09 and RMS-10) with other therapies (IVA/V or IVA). Mean HMGB1 and HMGB2 expression levels were approximately 10-fold higher than HMGA2 and HMGB3 or two times higher than HMGA1 (Figure 1). HMGB1 mRNA levels showed a significant negative Pearson correlation with OS (ρ = −0.714, *p* < 0.05) and a similar tendency with PFS (ρ = −0.626, *p* < 0.097; ρ = −0.752, *p* < 0.032 if we consider the PFS observed in RMS-05 after rechallenge of BOMP-EPI). HMGB1 mRNA levels in the patient with a complete response (RMS-05) were 3–10-fold lower than in the other patients (Figure 2a). No significant correlation was observed for HMGB2 and HMGA2 mRNA levels, which showed high variability in the different patients (Figure 2b,c).

**Figure 3:**
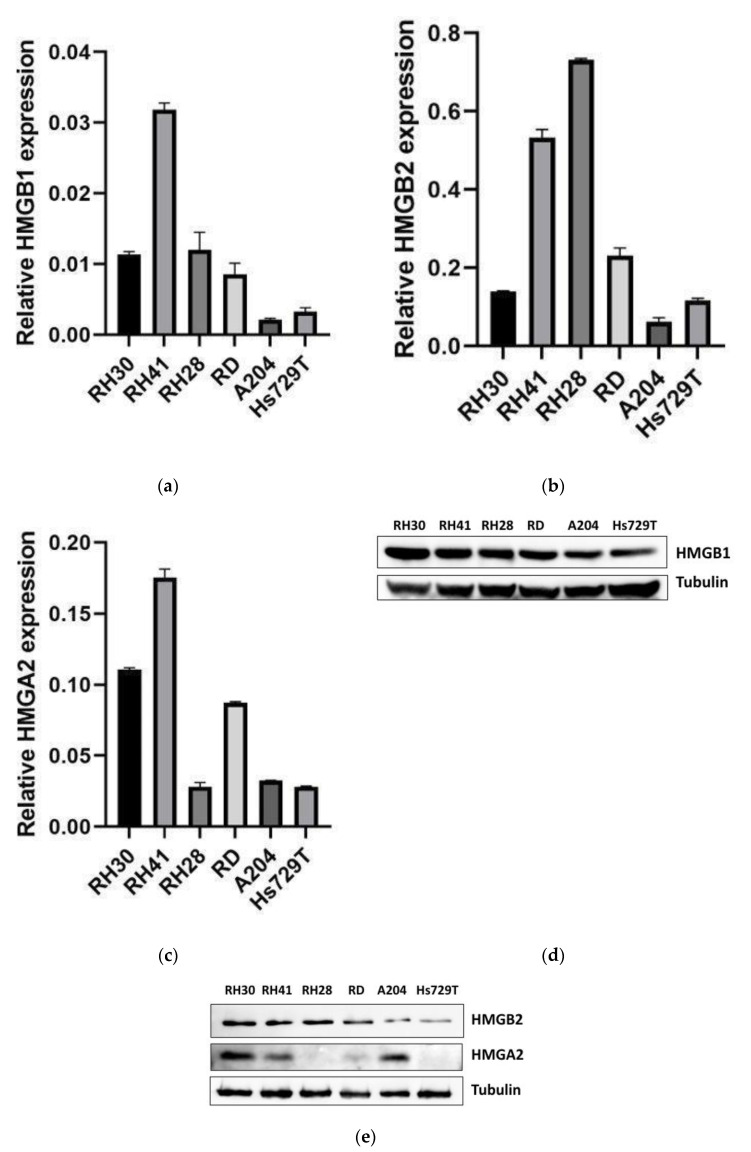
HMGB1, HMGB2 and HMGA2 expression in RMS cell lines. HMGB1 (**a**), HMGB2 (**b**) and HMGA2 (**c**) gene expressions in six RMS cell lines were analyzed by qPCR and normalized by GAPDH expression. RNA was extracted from each cell line 48 h after being seeded. HMGB1 (**d**), HMGB2 and HMGA2 (**e**) protein levels in RMS cell lines were analyzed by immunoblot using the specific antibodies for each protein and normalizing them by tubulin. The data from one of three independent experiments are shown. RH30, RH41 and RH28: alveolar rhabdomyosarcoma cell lines. RD, A204 and Hs729T: embryonal rhabdomyosarcoma cell lines.

HMGB1, HMGB2 and HMGA2 expression levels were analyzed in six RMS cell lines: three embryonal (RD, A204 and Hs729T) and three alveolar (RH30, RH41 and RH28). HMGB1 mRNA levels were 4–15-fold lower in A204 and Hs729T cell lines than in RH30, RH41, RH28 and RD cell lines. A204 and Hs729T cell lines also showed approximately two times less of HMGB1 protein than in RH30, RH41, RH28 and RD cell lines (Figure 3a,d). Moreover, A204 and Hs729T cell lines showed a slightly lower cisplatin IC50 (7.6–8.7 µM), together with RH28 versus RH41, RH30 and RD cell lines (9.7–11.8 µM), after 48 h of treatment (Table 4). HMGB2 mRNA levels were lower in A204 and Hs729T cell lines, which showed approximately 3–4-fold less HMGB2 protein levels than RH30, RH41, RH28 and RD cell lines (Figure 3b,e). HMGA2 mRNA levels were lower in RH28, A204 and Hs729T cell lines but RH28, RD and Hs729T cell lines showed 2–10-fold less HMGA2 protein levels, indicating no good correlation of mRNA with protein for A204 cell lines (Figure 3c,e).

Thus, low HMGB1 expression levels observed in patients with RMS with a better response to BOMP-EPI and in RMS cell lines with lower cisplatin IC50 could indicate that HMGB1 is a predictive biomarker of cisplatin response.

### 2.3. HMGB1 Knockdown Reduces Proliferation of RMS Cell Lines and Enhances Cisplatin Sensitivity

**Figure 4:**
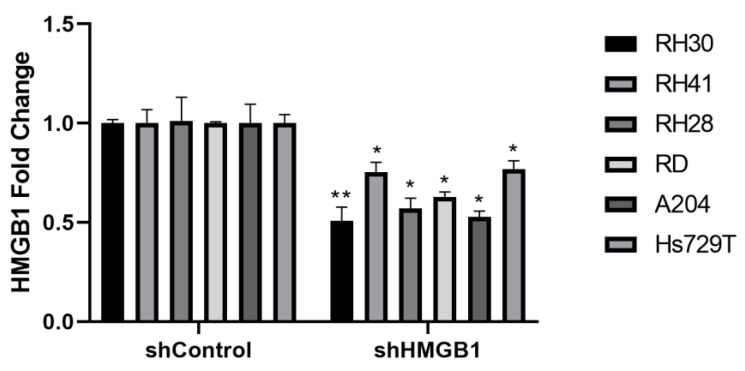
HMGB1 expression levels in RMS cell lines after HMGB1 knockdown. The HMGB1 gene expression in RH30, RH41, RH28, RD, A204 and Hs729T cell lines transduced with shHMGB1 lentivirus was compared against transduction with shControl lentivirus by qPCR, normalizing with GAPDH expression. * *p* < 0.05, ** *p* < 0.01, Student *t* test. RNA was extracted from each cell line 4 days after infection. The data from one of two independent experiments are shown. RH30, RH41 and RH28: alveolar rhabdomyosarcoma cell lines. RD, A204 and Hs729T: embryonal rhabdomyosarcoma cell lines.

**Figure 5:**
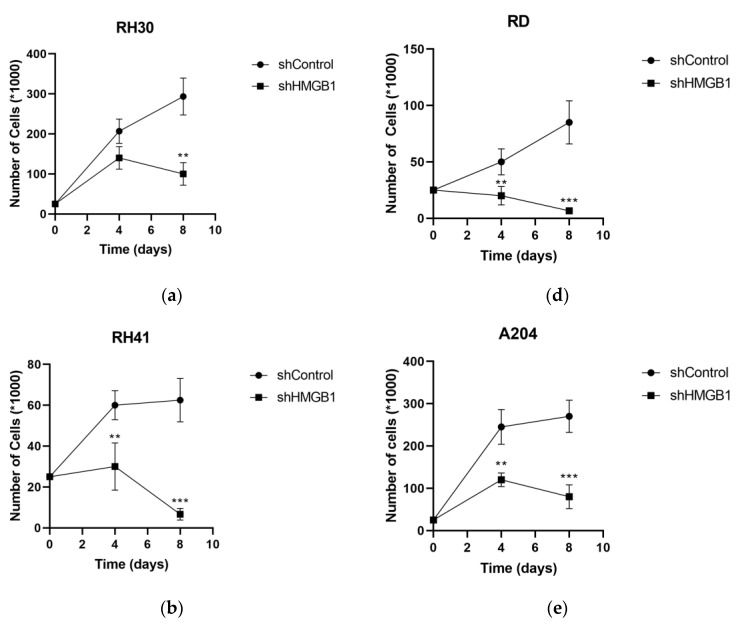

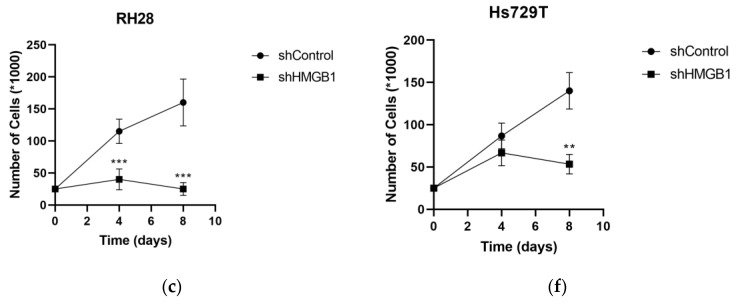
Proliferation of RMS cell lines after HMGB1 knockdown. The number of cells for the RH30 (**a**), RH41 (**b**), RH28 (**c**), RD (**d**), A204 (**e**) and Hs729T (**f**) cell lines was counted after 4 and 8 days of transduction with shHMGB1 lentivirus and compared against transduction with shControl lentivirus. ** *p* < 0.01, *** *p* < 0.001, Student *t* test. The data from one experiment are shown counting cells for quadruplicate in each point. RH30, RH41 and RH28: alveolar rhabdomyosarcoma cell lines. RD, A204 and Hs729T: embryonal rhabdomyosarcoma cell lines.

HMGB1 was downregulated in the six RMS cell lines by transduction with lentivirus harboring shRNA against HMGB1 (shHMGB1) or nontargeting shRNA for control (shControl). HMGB1 mRNA levels were decreased by 40%–50% in RH30, RH28, RD and A204 cell lines and 25% in RH41 and Hs729T cell lines transduced with shHMGB1 lentivirus versus transduction with shControl lentivirus (Figure 4). All the RMS cell lines showed a strong decrease of 62%–94% in proliferation after eight days of HMGB1 downregulation (Figure 5), indicating that HMGB1 is necessary for the proliferation of RMS cell lines.

**Figure 6:**
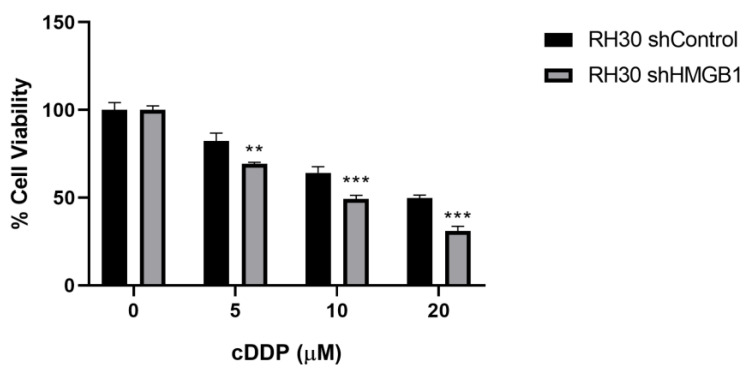
Sensitivity to cisplatin treatment in RH30 cell lines after HMGB1 knockdown. RH30 transduced with shHMGB1 lentivirus were treated with cisplatin (cDDP) at 5, 10 and 20 µM over 48 h and percentage of cell viability was compared versus RH30 transduced with shControl lentivirus. ** *p* < 0.01, *** *p* < 0.001, Student *t* test. The data from one of two independent experiments are shown.

RH30 cell lines with stable downregulation of HMGB1 (RH30 shHMGB1) after transduction with shHMGB1 lentivirus were selected by puromycin treatment for 2 weeks as well as for transduction with shControl lentivirus (RH30 shControl). RH30 shHMGB1 cell lines were treated with cisplatin at 5, 10 and 20 µM over 48 h, showing additional sensitivity to a cisplatin dose of 20% versus RH30 shControl with the different cisplatin doses (Figure 6).

## 3. Discussion

Treatment with platinum-based chemotherapy in this retrospective series of adult patients with advanced rhabdomyosarcoma suggests activity from this regimen, with an overall response rate (ORR) of 60% and a median PFS exceeding 8 months. Toxicity was mainly hematologic, as expected, with two patients withholding therapy because of side effects, but there were no toxic deaths. Our series has some limitations, its retrospective nature and its limited size being the major issues. Consequently, our findings and conclusions are hypothesis generating and should be validated in larger prospective series. However, data regarding the systemic therapy of adult patients with advanced or relapsed rhabdomyosarcoma is scarce in the literature. The management of adult patients with RMS remains controversial owing to their bare representation in clinical trials, but it seems that they do benefit from the same pediatric regimes [26]. There is no standard therapy for relapsed or metastatic disease, so chemotherapy based on anthracyclines and alkylating agents remains as the first-line option after several decades. The outcome in the metastatic setting seems to be influenced by the histologic subtype, where patients with alveolar RMS were those who had the worst prognosis when compared with embryonal RMS. In our series, both subtypes seemed to benefit equally from BOMP-EPI with similar median PFS, though in our series, patients with embryonal RMS had a longer OS, which is in line with previously reported data on adult patients [27]. Improving the poor prognosis of patients with relapsed or metastatic rhabdomyosarcoma remains an unmet need, and new therapeutic alternatives are desperately needed. Previously published data on second lines in RMS, mainly from pediatric series, come from small phase II trials or from retrospective series [26,28,29,30,31,32,33,34,35] (Table 5). Platinum-based regimens have scarcely been explored in advanced RMS [10,12,13,28,36], but the available data suggest activity from these combinations, with a wide range of reported response rates, between 28% and 100% (Table 5). However, outcomes in these series are difficult to analyze because they are heterogeneous in population and include only children or other also young adults, with different subtypes of sarcoma and reported outcomes. There are also some case reports of relapsed embryonal RMS describing the activity of cisplatin- based regimens and showing complete response [37]. However, to the best of our knowledge, this is the largest series reporting on the activity of platinum-based chemotherapy in adult patients with rhabdomyosarcoma. 

HMGB1, HMGB2 and HMGA2 genes have been analyzed in tumors of these patients with RMS because they could affect DNA-platinum adduct repair and, in consequence, their sensitivity to this therapy. HMGB1 mRNA levels showed a significant negative correlation with OS and the same tendency for PFS, which is especially interesting in the patient with complete response who showed the lowest HMGB1 mRNA levels and the highest PFS and OS of this RMS series. The A204 and H729T cell lines, which are two of the most sensitive RMS cell lines to cisplatin treatment also showed the lowest levels of HMGB1 mRNA and protein levels. HMGB1 knockdown in the six RMS cell lines assayed showed a strong reduction of the proliferation, indicating HMGB1 could be a new target for the treatment of RMS. On the other hand, the overexpression of HMGB1 has been associated with proliferation, angiogenesis, the evasion of programmed cell death, invasion and metastasis. Moreover, HMGB1 can be released to the extracellular milieu and acts as a damage-associated molecular pattern (DAMP) molecule with function in the inflammatory response [38,39]. Our data also show that HMGB1 downregulation enhances RMS sensitivity to cisplatin, indicating that low HMGB1 expression could be a predictive factor of benefit to therapy with cisplatin. HMGB1 overexpression has been associated with resistance to cisplatin treatment in human cancer cervical cells [40], non-small-cell lung cancer [41], neuroblastoma [42] and osteosarcoma [23,43], by mechanisms of cell autophagy, a fundamental lysosomal process that confers stress tolerance and inhibits apoptosis. New research is necessary to identify whether HMGB1 induces cisplatin resistance by mechanisms of cell autophagy or by binding to cisplatin adducts and protecting them from being repaired, as has been described for other HMG proteins [20,21].

HMGB2 and HMGA2 mRNA levels did not correlate with OS and PFS in these patients with RMS. However, the lowest levels of HMGB2 mRNA and protein were also observed in A204 and H729T RMS cell lines, and the lowest levels of HMGA2 mRNA and protein were observed in RH28 and H729T RMS cells. Hs729T, RH28 and A204 cell lines are the most sensitive RMS cell lines to cisplatin among the six RMS cell lines analyzed. HMGB2 overexpression has been associated with resistance to cisplatin in head and neck squamous cell carcinoma [44], as well as tumor aggressiveness and prognosis of hepatocellular carcinoma [45]. HMGA2 is a critical regulator in the development of some tumors, including sarcomas and rhabdomyosarcomas, generally related to bad prognoses [46,47,48,49,50,51]. On the other hand, the association of HMGA2 with cisplatin sensitivity has been controversial in different tumors; while in some cancers, HMGA2 levels have been related with cisplatin sensitivity, in other tumors, it has been associated with resistance to this drug [52,53,54,55,56]. 

In conclusion, in our experience, platinum-based regimens are active in adult patients with relapsed and advanced rhabdomyosarcoma, with manageable expected toxicity. These observations deserve their own explorations in a prospective study. Moreover, low HMGB1 expression levels could be used as predictive factors of good responses to BOMP-EPI treatment.

## 4. Materials and Methods

### 4.1. Rhabdomyosarcoma Cell Lines

Embryonal RMS cell line RD was purchased from Merck (Rahway, NJ, USA), embryonal RMS cell line A204 (ATCC HTB-82) and Hs729T (ATCC HTB-153) were purchased from ATCC (Manassas, VA, USA) and alveolar RMS cell line RH41 was purchased from DSMZ (Braunschweig, Germany, DSMZ ACC-592). Alveolar RMS cell lines RH30 and RH28 were kindly provided by Dr. Amancio Carnero (Institute of Biomedicine of Seville, CSIC, US, HUVR; Seville, Spain) and Dra. Soledad Gallego (Hospital Vall d’Hebron, Barcelone, Spain), respectively.

RD, RH30 and Hs729T were cultured in DMEM medium (Gibco, Thermo Fisher Scientific, Waltham, MA, USA). RH41 and RH28 were cultured in RPMI medium (Gibco, Thermo Fisher Scientific, Waltham, MA, USA). A204 was cultured in McCoy’s 5A medium (Gibco, Thermo Fisher Scientific, Waltham, MA, USA). All cell culture mediums were supplemented with 10% FBS (Gibco, Thermo Fisher Scientific, Waltham, MA, USA), 100 units/mL penicillin and 100 µg/mL streptomycin (Sigma-Aldrich, St. Louis, MO, USA). Additionally, DMEM medium was supplemented with 100 µM sodium pyruvate (Sigma-Aldrich, St. Louis, MO, USA) and 1 mM MEM NEAA (Gibco, Thermo Fisher Scientific, Waltham, MA, USA).

All cell lines were incubated in a 5% CO_2_ atmosphere and at 37 °C, and they were regularly tested for mycoplasma contamination.

#### Rhabdomyosarcoma Cell Line Transduction

Each RMS cell line was seeded in MW24 at 25,000 cells/well and was incubated with lentivirus, which expresses HMGB1 (shHMGB1) or control (shControl) short hairpin RNAs at a MOI = 1 (Santa Cruz Biotechnology, Dallas, TX, USA, sc-37982-V and sc-10808) plus polybrene (Santa Cruz Biotechnology, Dallas, TX, USA) at 5 µg/mL overnight. The number of cells was counted with trypan blue in a Neubauer chamber after 4 and 8 days of the transduction. Cells with stable expression of shHMGB1 or shControl were selected by treatment with puromycin at 0.25 µg/mL.

### 4.2. Patients

Patients diagnosed with RMS (excluding pleomorphic histologic subtype) from August 1994 to November 2015 in Hospital Universitario Virgen del Rocío (Seville) and Hospital Universitario Son Espases (Palma de Mallorca) were retrospectively reviewed. Patients were eligible for this analysis if they had more than 16 years at diagnosis and they had been treated with platinum-based regimes, specifically BOMP-EPI (bleomycin, vincristine, methotrexate and cisplatin alternating with etoposide, cisplatin and ifosfamide) [57] (Table 6). Data regarding clinical and histopathological characteristics, therapy and survival were collected. The risk group was defined on the basis of the Intergroup Rhabdomyosarcoma Study (IRS) and also on the European Pediatric Soft Tissue Sarcoma Study Group (EpSSG) classifications. All diagnoses were confirmed by a pathologist with expertise in sarcomas. Radiological responses were evaluated using RECIST 1.1 [58]. Toxicity was evaluated according to CTC 4.0. Statistical analysis was conducted using SPSS version 25. The Kaplan–Meier method was used for time-to-event variables. Progression-free survival (PFS) to BOMP-EPI was defined as the period between the first dose of therapy and evidence of radiological progression or death of any cause. Ethics Committee Approval was obtained for this study (study GEI-SAR-2015-01). All patients signed informed consent for chemotherapy.

### 4.3. Gene Expression Assay in Tumor Samples

Paraffined tumor samples were obtained from Hospital Universitario Virgen del Rocío and Hospital Universitari Son Espases, after receiving the patient’s informed consent for the study. FOXO1 rearrangement was confirmed by FISH in all RMS cell lines and BOMP-EPI-treated tumor samples. Gene expression analysis was performed with HTG EdgeSeq technology (HTG Molecular Diagnostics, Tucson, AZ, USA), using the EdgeSeq Oncology Biomarker Panel (OBP) for quantification of 2549 human RNA transcripts (https://www.htgmolecular.com/assays/obp, accessed on 12 September 2020) related to tumor biology. RNA was extracted from paraffined tumor samples with at least 70% of tumor area or from selected tumor area by macrodissection in cases with less than 70% of tumor area or more than 20% of necrosis. RNA-Seq libraries were synthesized in the HTG EdgeSeq system by using the HTG EdgeSeq chemistry and following the specific instructions and recommendations for sequencing with the Illumina technology. RNA-Seq libraries were cleaned with Agencourt AMPure XP (Beck-man Coulter, Beverly, MA, USA) and quantified with the KAPA Library Quantification kit (Roche, Basel, Switzerland) by qPCR, according to the manufacturer’s instructions. Library denaturation was performed by adding the first 2 N NaOH to the library, followed by the addition of 2 N HCl. The PhiX was spiked in at a 5% (concentration of 12.5 pM).

One demultiplexed FASTQ file per sample was retrieved from the sequencer for data processing. The HTG EdgeSeq host software performed the alignment of the FASTQ files with the probe list, the results were parsed, and the output was obtained as a read count matrix. The HTG EdgeSeq was run in the VERIP service laboratory of HTG in Tucson (HTG MolecularDiagnostics, Tucson, AZ, USA).

### 4.4. Cisplatin Treatment

A total of 2500 or 5000 cells/well were seeded in 96 well plates and were treated 24 h later with 0.1, 1, 10 and 100 µM of cisplatin (Sigma-Aldrich) or drug vehicle. After 72 h, 20 µL of Cell Titer 96 AQueous One Solution Cell Proliferation Assay (MTS) (Promega, Madison, WI, USA) was added to each well, and absorbance was measured at 490 nm in iMark microplate absorbance reader (Bio-rad, Hercules, CA, USA). The IC50 of each RMS cell line was calculated with a nonlinear regression. 

A total of 5000 cells/well of RH30 shControl and shHMGB1 were seeded in 96 well plates and treated 8 h later with 5, 10 and 20 µM of cisplatin (Sigma-Aldrich) or drug vehicle. After 48 h, 20 µL of Cell Titer 96 AQueous One Solution Cell Proliferation Assay (MTS) (Promega, Madison, WI, USA) was added to each well, and absorbance was measured at 490 nm in iMark microplate absorbance reader (Bio-rad, Hercules, CA, USA).

### 4.5. RNA Extraction and RT-qPCR

RNA was extracted from RMS cell lines with the RNA PureLink RNA Mini kit (Invitrogen, Carlsbad, CA, USA), quantified with the NanoDrop One C spectrophotometer (Thermo Fisher Scientific, Madison, WI, USA) and reverse transcribed to cDNA using the High-Capacity Reverse cDNA Transcription kit (Applied Biosystems, Thermo Fisher Scientific, Foster City, CA, USA). Expression levels of each gene were quantified by RT-qPCR in an ABI Prism 7900HT real-time PCR system by using the TaqMan Universal PCR Master Mix (Applied Biosystems, Thermo Fisher Scientific) and the specific TaqMan Gene Expression Assays (Applied Biosystems) HMGA2 (Hs00171569_m1), HMGB1 (Hs01923466_g1), HMGB2 (Hs01127828_g1) and GAPDH (Hs03929097_g1) as a housekeeping gene for data normalization.

### 4.6. Western Blotting

Cell lysis and protein extraction were carried out using the RIPA buffer (1 M Tris-HCl pH 8 (PanReac AppliChem, ITW Reagents), 0.5 M EDTA (Thermo Fisher Scientific), Triton X-100 (Sigma-Aldrich), 10% sodium deoxycholate (Sigma-Aldrich), 10% SDS (Sigma-Aldrich) and 3 M NaCl (Thermo Fisher Scientific)), supplemented with protease and phosphatase inhibitor cocktail (Sigma-Aldrich). Further, 20 µg of protein from each sample were separated by SDS-PAGE and transferred to a 0.2 µm pore-size nitrocellulose membrane (Bio-Rad). Membranes were blocked for 1 h with 5% BSA (PanReac AppliChem, ITW Reagents) in 1x TBS-T (0.1% Tween20, Bio-Rad) and incubated with specific antibodies HMGA2 (Proteintech, 20795-1-AP), HMGB1 (Abcam, ab18256), HMGB2 (Proteintech, 14597-1AP) and ɑ-tubulin (Sigma-Aldrich, T9026) overnight at 4 °C. Then, membranes were washed with 1x TBS-T and incubated with rabbit antimouse IgG (Sigma-Aldrich) or goat antirabbit IgG (Abcam, Cambridge, UK), both conjugated with peroxidase. HRP substrate (GE Healthcare, Life Sciences) was used for chemiluminescent detection, and image acquisition was performed using a Chemidoc Imaging System (Bio-Rad).

## Figures and Tables

**Table 1:** Demographic and clinical characteristics from the series.

Patient ID	Gender	^1^ Age (Years)	Histologic Subtype	Pathology Details	Location	^2^ IRS Group	Risk Group	^3^ Upfront Syst. Therapy	Surgery
RMS-01	F	19.9	Alveolar	Myo, Des FOXO+	Limb	IV	Very high risk	IVADo/IVA	Y (R1)
RMS-02	F	17.2	Alveolar	Myo, Act, V	Limb	I	High risk	IVA	Y (R0)
RMS-03	M	21.6	Alveolar	Myo, Des	Head & Neck	IIb	Very high risk	IVA/V	Y (R0)
RMS-04	M	16.7	Embryonal	Myo, Des	Heart	IV	High risk	EPI-IFOS	Y (R0)
RMS-05	M	27.4	Embryonal	Des, Act	Pelvis	IV	High risk	BOMP/EPI	Y (R0)
RMS-06	F	20.8	Embryonal	Des, Act	Head & Neck	IV	High risk	BOMP/EPI	Y (R0)
RMS-07	F	42.2	Alveolar	Des, Act	Head & Neck	IV	Very high risk	BOMP/EPI	N
RMS-08	M	20.9	Alveolar	Myo, Act, V. FOXO+	Head & Neck	III	Very high risk	VAC/IE	Y (R2)
RMS-09	F	20.8	Alveolar	Myo, FOXO−	Head & Neck	IIIa	High Risk	VAC/IE	Y (R0)
RMS-10	F	44.1	Spindle cell	Myo, Des. FOXO−	Head & Neck	IIIa	High Risk	IVADo	Y (R1)

^1^ Age at diagnosis; ^2^ Intergroup rhabdomyosarcoma study groups: I—complete resection of primary tumor; II—microscopic residual disease; III—macroscopic residual disease; IV—metastasis from disease onset. ^3^ Upfront systemic therapy; F—female; M—male; Myo—myogenin; Des—desmin; Act—actin; V—vimentin; FOXO—evidence of FOXO rearrangement; IVADo—ifosfamide, vincristine, actinomycin and doxorubicin; IVA—ifosfamide, vincristine, actinomycin; VAC—vincristine, doxorubicin and cyclophosphamide; IE—ifosfamide and etoposide; EPI/IFOS—epirubicin and ifosfamide; BOMP-EPI—bleomycin, vincristine, methotrexate and cisplatin plus etoposide, cisplatin and ifosfamide; Y—Yes; N—No.

**Table 2:** Treatment and toxicity.

Characteristics	N (%)
Previous lines of systemic therapy:	
0	3 (30)
1	5 (70)
2	2 (20)
Previous chemotherapy:	
IVADo/IVA	4 (60)
VAC/IE	2 (20)
EPI/IFOS	1 (20)
Median of number of cycles (range) of BOMP-EPI	2.5 (1–8)
Toxicity:	
• Hematologic:	
Grade 3-4 neutropenia	7 (70)
Neutropenic fever	2 (20)
G3-4 Anemia	2 (20)
G3-4 thrombocytopenia	1 (10)
• Non-hematologic:	
Grade 2 neuropathy	1 (10)
Grade 2 hearing impairment	1 (10)
Grade 3 mucositis	1 (10)
• Toxic deaths	0

IVADo—ifosfamide, vincristine, actinomycin and doxorubicin; IVA—ifosfamide, vincristine, actinomycin; VAC—vincristine, doxorubicin and cyclophosphamide; IE—ifosfamide and etoposide; EPI/IFOS—epirubicin and ifosfamide; BOMP-EPI—bleomycin, vincristine, methotrexate and cisplatin plus /etoposide, cisplatin and ifosfamide.

**Table 3:** RECIST Response. Patient’s outcome on BOMP-EPI.

Patient	Line	Best RECIST Response	PFS (Months)	OS (Months)
RMS-01	2	SD	4.9	10.8
RMS-02	2	PR	8.5	24.7
RMS-03	2	SD	24.3	30.5+
RMS-04	2	SD	8.9	18.3
RMS-05	1	PR	28.9	258+
RMS-06	1	PR	6.2	26.2
RMS-07	1	PR	11.3	35.4
RMS-08	2	PD	1	1
RMS-09	3	PR	3.5	3.8
RMS-10	3	PR	8.3	15.7+
RMS-02 *	3	PR	15.8	16.2
RMS-04 *	3	SD	9.4	9.4
RMS-05 *	2	CR	229+	258+

PFS: progression-free survival; OS: overall survival; CR: complete response; PR: partial response; SD: stable disease; PD: progressive disease; +: free of progression in last follow-up; * These patients received rechallenges of BOMP-EPI at progression.

**Table 4:** Cisplatin IC50 in RMS cell lines.

Subtype	Cell Line	cDDP IC50 (µM)
ERMS	A204	7.6
ARMS	RH28	7.8
ERMS	Hs729T	8.7
ARMS	RH41	10.3
ARMS	RH30	9.7
ERMS	RD	11.8

ERMS: embryonal rhabdomyosarcoma; ARMS: alveolar rhabdomyosarcoma.

**Table 5:** Published evidence of second-line systemic therapy in rhabdomyosarcoma.

Regimen	Type of Study	N	Setting	RR	PFS (Months)	Toxicity	Ref
Vinorelbine	Phase II	33 (13 RMS)	Pediatric sarcoma patients	50%	3.5 (all the series)	63% G3-4 neutropenia	[29]
Vinorelbine-Cyclophosphamide	Phase II	117 (50 RMS)	Pediatric and <25 and adults relapsed tumors	36%	NR	38% G3-4 neutropenia	[26]
Cyclophosphamide-Topotecan	Retrospective	15 (6 RMS)	Adult relapsed sarcoma patients	33% (2/6)	2.5 (all the series)	47% hematologic toxicity	[30]
Cyclophosphamide-Topotecan	Phase II window trial	61	Pediatric metastatic RMS (1st line)	47%	NR (3-y DFS: 10%)	G3-4: leucopenia 52%, anemia 37%	[31]
Cyclophosphamide-Topotecan	Phase II	91 (15 RMS)	Pediatric relapsed tumors	66% (10/15 RMS)	NR	G3-4: leucopenia 53%, anemia 27%	[32]
Vincristine-Irinotecan	Phase II window trial (2 regimens)	92	Pediatric relapsed RMS	26–37%	NR (1y-FFS 37 and 38%)	≥G3: 50–66%	[33]
Vincristine-temozolomide-irinotecan	Retrospective	19	Pediatric relapsed RMS	0	PFS-3 months: 23%	NR	[34]
Topotecan-carboplatin	Phase II	38	Pediatric relapsed RMS	28%	5-y PFS 14%	63% G4 hematologic	[13]
Cisplatin-etoposide	Phase II	27 (21 RMS)	Pediatric relapsed tumors	33% (7/21)	NR	NR	[12]
Ifosfamide-carboplatin-etoposide (ICE)	Phase I and II trials analysis	97 (27 RMS)	Pediatric and adolescent relapse sarcoma	66% (RMS)	NR	G3-4 hematologic: 100%	[36]
Vincristine, ifosfamide and doxorubicin (VIA)/etoposide, ifosfamide and cisplatin (VIP)	Retrospective	6	Adult patients (advance disease)	100%	NR	NR	[28]
Several regimenes	Retrospective	49	Pediatric relapsed RMS		NR	NR	[10]
Carboplatin-epirubicin- vincristine ad ifosfamide-vincristine- etoposide (CEV/IVE)		15		73.3%			
Vincristine/irinotecan ± temozolomide (VI[T])		7		42.9%			
Gemcitabine-docetaxel	Retrospective	19 (5 RMS)	Pediatric relapsed sarcoma	40% (2/5)	2 (all series)	G3-4 toxicity 74%	[35]

RR: response rate; PFS: progression-free survival; NR: not reported; DFS: disease-free survival; FFS: failure-free survival; RMS: rhabdomyosarcoma.

**Table 6:** Details of BOMP-EPI regimen.

BOMP	EPI
Day 1: Vincristine 2 mg bolus iv Methotrexate 100 mg/m^2^ iv in 20′ Methotrexate 200 mg/m^2^ in 12 h infusion	Days 1–4: Cisplatin 25 mg/m^2^ in 30′ Etoposide 120 mg/m^2^ in 90′ Ifosfamide 1300 mg/m^2^ in 60′ MESNA rescues as per protocol
Day 2: Bleomycin 30 mg in 12 h infusion	
Day 3: Cisplatin 100 mg/m^2^ in 30′	

## Data Availability

Data are available in a private area of the sponsor’s website (www.grupogeis.org, accessed on 1 November 2022) and will be available beginning 3 months and ending 5 years after publication of the initial study results. Data requests should be sent to secretaria@grupogeis.org.
